# Dairy products and bone health

**DOI:** 10.1007/s40520-021-01970-4

**Published:** 2021-09-07

**Authors:** René Rizzoli

**Affiliations:** Service of Bone Diseases, Geneva University Hospitals and Faculty of Medicine, 1211 Geneva, Switzerland

**Keywords:** Growth, Bone mineral density, Osteoporosis, Fracture, Nutrition, Probiotics, Protein, Calcium, Fermented dairy products

## Abstract

Bone mineral mass, geometry and microstructure, hence determinants of fracture risk, result bone accrual during growth and bone loss later in life. Peak bone mass, which is reached by the end of the second decade of life, is mainly determined by genetic factors. Among other factors influencing bone capital, dietary intakes, particularly calcium and protein, play a significant role in peak bone mass attainment. Both nutrients are provided in dairy products, which accounts for 50–60% and 20–30% of the daily calcium and protein intakes, respectively. Children avoiding dairy products are at higher risk of fracture, as are adults or older individuals following a diet devoid of dairy products, like vegans. Various intervention trials have shown some beneficial effects of dairy products on bone capital accumulation during growth and on bone turnover in adults. In observational studies, dairy products intake, particularly the fermented ones, which also provide probiotics in addition to calcium, phosphorus and protein, appear to be associated with a lower risk of hip fracture.

## Introduction

Skeleton functions include body support, internal organ protection, mineral homeostasis and a role in acid–base regulation. Bone strength depends on bone mass, geometry, microstructure and material level properties. Maximal bone capital, i.e. peak bone mass, is reached by the end of the second decade of life, and, under usual conditions, allows us to successfully resist to a mechanical overload [1]. Between 60 and 80% peak bone mass variance are explained by genetic factors. Environmental factors can modify the influence of genetic factors, impair bone mass accrual, alter bone turnover and/or bone strength, and increase thereby fracture risk. During childhood and adolescence, height gain and bone mineral mass accrual are following a track which is genetically determined [1, 2]. Any nutritional insufficiency can alter bone growth and move the trajectory towards a less favorable track, and lead thereby to a lower peak bone mass. Nutrients such as calcium, phosphorus and protein are major nutritional determinants of bone mass accrual. These nutrients are combined in dairy products. Indeed, one liter of cow milk provides 1200 mg/l, 1150 mg/l phosphorus, 32–35 g/l protein, i.e. casein and whey protein, which also contains a series of cellular growth factors, together with calories, trace elements and vitamins (Table 1). Whey proteins are faster digested and absorbed than casein. Despite variations of milk composition according to cow breed, seasons and food, commercially available milk is usually standardized, and sometimes fortified with vitamin D in a few countries. Depending on the species, the nutrients content can considerably vary (Table 2). The macronutrient concentrations of some plant drinks may be similar to that of cow milk, such as for instance protein content. However, plant-based alternatives require the addition of mineral salts and of carbohydrates to reach concentration of calcium and of calories similar to cow milk [3] (Table 3). However, the nutritional quality of most plant drinks is markedly different. If cow milk is replaced by non-fortified and non-supplemented plant drinks, consumers may risk various deficiencies, thus children and adolescents receiving exclusively such plant drinks may be at risk of severe metabolic disturbances [4]. Indeed, the supplemental carbohydrate content cannot be considered as part of a healthy diet. Except for soy drink, the other preparations cannot bear the name of milk. Animal sources of protein tend to be more easily digested, and the distribution of essential amino acids is considered to better fit human requirements, particularly for muscle and bone formation [5].

**Table 1:** Bone nutrient content per 100 g of selected dairy foods

Dairy food	Calcium (mg)	Potassium (mg)	Phosphorus (mg)	Protein (g)
Milk, full-fat 3.7%	119	151	93	3.3
Milk, skimmed	122	156	101	3.4
Yoghurt, plain low-fat*	183	234	144	5.3
Yoghurt, fruit low-fat	169	216	133	4.9
Cheddar cheese	721	98	512	24.9
Cottage cheese, non-fat	86	137	190	10.3
Ice cream, soft serve, chocolate	131	177	116	4.1

Data are from the USDA National Nutrient Database for Standard Reference, release 26. 2013

Available at: http://ndb.nal.usda.gov/ndb/nutrients/index

**Table 2:** Chemical constituents of human, cow, goat, sheep, camel and buffalo milk

Milk	Protein g/100 g	Lactose g/100 g	Fat g/100 g	Calcium mg/100 ml	pH
Human	1.25	6.95	3.20	32.00	7.20
Cow	3.40	4.80	3.75	112.00	6.60
Goat	3.30	4.40	3.90	130.00	6.60
Sheep	6.35	5.00	6.90	197.50	6.60
Camel	2.95	4.30	3.60	94.40	6.50
Buffalo	4.52	4.80	7.94	173.4	6.77

From [80]

**Table 3:** The nutritional profile of cow´s milk and plant-based alternatives, with and without fortification

	Nutritional content per 100 ml of beverage							
	Milk	Soy drink	Soy drink, fortified	Almond drink	Rice drink	Rice drink, fortified	Oat drink	Oat drink, fortified
Energy, kcal	64	55	45	47	50	54	50	45
Energy, kJ	268	230	188	197	209	226	209	188
Protein, g	3.4	3.1	3.3	0.7	0.1	0.1	0.6	0.5
Total lipid, g	3.5	2.3	1.9	2.2	0.9	0.9	1	1.6
Carbohydrate, g	4.9	5.3	3.6	5.9	10.3	11.4	9.7	7.2
Vitamin A, RE	35.3	0	0	0	0	0	0.34	0
Vitamin B2, mg	0.18	0.01	0.02	0.02	0	0	0.01	0.01
Vitamin B12, µg	0.39	0	NA	NA	NA	NA	NA	NA
Calcium, mg	119	9.86	74.5	8.8	1.85	84.3	6.56	126
Zinc, mg	0.36	0.25	0.28	0.11	0.03	0.05	0.41	0.08
Iron, mg	0.02	0.45	0.5	0.12	0.01	0.23	0.03	0.44
Iodine, µg	16.5	1.3	9.35	0.89	1.04	2.5	0.418	5.9
Phosphorus, mg	91	44.1	41.5	14.3	7.39	28	13.2	16.9

Data from the Danish National Food Institute, food D Frida food data. DTU Fødevareinstituttet. https://frida.fooddata.dk/

*RE* retinol equivalents, *NA* not assessed

Dairy products are consumed by humans since millennia, as indicated by processed dairy residues detected in pottery vessels found in Dalmatian cost or in Anatolia, and going back to 6000 BC [6, 7]. Consumption of cow, sheep or goat milk is confirmed by finding dairy protein in dental calculus from northeastern Africa at least 6 millennia ago [8]. The first mention in the literature of cheese making is likely in the Odissey of Homer (chant IX), written around 750 BC. Fermented dairy products like cheese and yoghurts have allowed the preservation, the transport and an easier digestion of milk.

The role of the consumption of dairy products, which are a complex combination of macronutrients and micronutrients, in adult bone homeostasis is still debated. Indeed, whilst the natural function of milk and dairies is to ensure normal growth of young mammals, the importance for bone health of its nutrients content and of dairy products as foods in young adults and in the oldest old to meet calcium and protein requirements is still not fully appreciated. In this narrative review, the effects on bone health of the main nutrients present in dairy products and of whole dairy foods are summarized and discussed.

## Literature search strategy

A literature search was conducted using MEDLINE database. Relevant observational studies and randomized controlled trials were selected using a combination of keywords including bone growth, osteoporosis, bone remodeling, BMD, BMC and fracture as outcomes; and dietary calcium and protein, milk, cheese and dairy products as explanatory variables. Additional studies were identified by an extensive manual search of bibliographic references in original papers and reviews. Abstracts and non-English papers were not included. Particular attention was given to finding randomized controlled trials. However, part of the information collected and presented is derived from observational studies.

## Dietary calcium intake and bone growth

In controlled intervention trials, milk ultrafiltrate calcium supplements increased peripheral skeleton bone mineral content in both prepubertal girls and boys [1]. These effects are attributed to lower bone turnover thus reduced resorption cavities. A meta-analysis including intervention trials comparing calcium supplements to a placebo has concluded to a favorable effect of calcium on peripheral skeleton, persisting at least 18 months after calcium discontinuation [9]. During a long-term follow-up up to adulthood of a cohort of healthy girls, having participated at the age of 8 years to a trial with 850 mg of calcium supplement per day of milk origin, leading to a doubling of the spontaneous intake, a persistent effect was observed in those girls with a menarcheal age below the group median [10]. However, there is poor evidence that calcium intake during childhood and adolescence would be associated with fracture risk later in life.

## Dietary phosphate

Adequate phosphate supply is required for cartilage and osteoid tissue mineralization [11]. Phosphate wasting syndromes are associated with impaired growth and fragility fractures [12]. A normal usual diet provides sufficient amounts of phosphate in most circumstances, so that phosphate deficiency from dietary origin is unlikely. Phosphate is found in high amounts in protein containing foods such as dairy products (1150 mg/l of milk and 500 mg/100 g of Swiss cheese), meat, and also in grains, beans, lentils and nuts. Recommended Dietary Allowance is 1250 mg/day for adolescents during growth and 700 mg/day for adults. Under normal conditions, 60–70% of dietary phosphate is absorbed. The low BMD in older women associated with colas beverages seems to be rather mediated by milk displacement, since 1 l cola contains 170 mg of phosphorus, thus far less than 1 l milk [13].

## Dietary protein intake and bone growth

In a prospective longitudinal observational study over 4 years, with an annual record of nutritional intakes [14], bone size, bone mass and an estimation of bone strength were positively correlated to dietary protein intake. However, there is presently no randomized intervention trial assessing whether this correlation is an association only or reflects a causal relationship. Liver IGF-I production is stimulated by dietary protein, particularly by aromatic amino acids [15]. IGF-I increases longitudinal and radial bone growth [16]. By enhancing renal tubular reabsorption of phosphate and the renal synthesis of calcitriol, hence stimulating intestinal absorption of calcium and phosphate, IGF-I contributes to warranting an optimal mineral concentration for mineralization of newly deposited cartilage and osteoid matrix [16].

## Dairy products and bone growth (for review see [17, 18])

Dairy products provide about 50–60% of calcium intake and 20–30% of protein intake during growth. Dairy products avoidance during childhood is a risk factor for fracture [19]. A diet devoid of dairies is associated with a 4.6-fold increase in fracture risk in girls between the age of 2 and 20 years [20]. Dairy products influence may intervene even before birth. Indeed, BMD of 6-year-old children was positively correlated with milk and calcium-rich foods consumed by the mother during pregnancy [21]. The first milk intervention trials took place in the 1920ies. Providing around 0.5 l milk to school children for 7 months increased height gain [22, 23]. Numerous trials have confirmed some benefits, even of small magnitude, of dairy products on bone mass accrual (Table 4). For instance, in a randomized controlled trial in 12-year-old girls, a pint of milk, corresponding to 568 ml, increased whole body mineral content, particularly in the lower limb, and IGF-I levels [24]. Compared to calcium supplements, cheese increased cortical bone mass [25, 26]. In 10–12-year old girls, calcium provided as cheese led to a higher bone gain as compared with calcium as pills [27]. An effect on bone modeling is likely since metacarpal bone diameter was higher in Chinese children receiving milk supplements than in controls [27]. Dairy products may thus influence bone mineral accrual through a remodeling process mediated by calcium and a modeling process through protein stimulated IGF-I production, favoring periosteal apposition.

**Table 4:** Effects of dairy products on bone in children and adolescents (controlled trials)

Study	Year	Number	Sex	Mean age (years)	Duration (months)	Intervention	Outcome	Main results Intervention—placebo changes
Baker et al. [81]	1980	581	F/M	8.0	21	Milk 190 ml/day	Height	Height: + 3% or + 2.93 mm
Cadogan et al. [24]	1997	82	F	12.2	18	Milk 568 ml/day	WB BMC; IGF-I	WB BMC: + 2.9% or + 37 g; IGF-I: + 10%
Chan et al. [82]	1995	42	F	11	12	Dairies	WB BMC; LS BMD	WB BMC: + 9.9%; LS BMD: + 6.6%
Cheng et al. [26]	2005	195	F	11.2	24	Cheese equivalent 1000 mg Ca	Tibia CTh; WB BMD	Tibia CTh: + 6%; WB BMD: + 2%
Du et al. [83]	2004	757	F	10.1	24	Calcium-fortified milk 330 ml/day	Height; size- adjusted WB BMC; WB BMD	Height: + 0.6%; size- adjusted WB BMC: + 1.2%;
								WB BMD: + 3.2%
Lau et al. [84]	2004	344	F/M	10.0	18	Milk powder equivalent to 1300 mg Ca	LS BMD; Hip BMD	LS BMD: + 1.4%; Hip BMD: + 1.1%
Leighton & Clark [23]	1929	1425	F/M	6–13	7	Milk 568 ml/day (426 ml if ≤ 7 yrs)	Height	Height: + 23.5%
Lu et al. [85]	2019	232	F/M	13.1	18	Milk powder fortified in Ca, equivalent to 20 g protein	IGF-I; WB, LS, Hip BMD	IGF-I: + 21%; BMD: no difference
Merrilees et al. [86]	2000	91	F	16	24	Milk equivalent to 1160 mg Ca	LS, FN, Trochanter BMD	Statistically significant differences in BMD changes from baseline
Orr [22]	1928	NR	M	5–14	7	Milk 568 ml/day (426 ml if ≤ 6 yrs)	Height	Height: + 21.3%
Vogel et al. [87]	2017	240	M/F	11.8	18	3 servings dairies/day	LS, Hip BMD; 4% tibia BMC	LS, Hip BMD: no difference in BMD changes from baseline; 4% tibia BMC: higher gain
Volek et al. [88]	2003	28	M	14	3	3 servings dairies/day with resistance training	Height; WB BMC; WB BMD	Height: + 0.8 cm; WB BMC: no difference;
Zhu et al. [27]	2005	606	F	10.1	24	Calcium-fortified milk 330 ml/day	Metacarpal outer diameter, CTh	Metacarpal outer diameter: + 1.2%; CTh: + 5.7%
							Metacarpal medullary diameter	Metacarpal medullary diameter:—6.7%
Zhu et al. [89]	2008	345	F	10.1	24	Calcium-fortified milk 330 ml/day	Size-corrected WB BMD	Size-corrected WB BMD: + 3.6–5.8%

+ Statistically significant greater change in the intervention group.  −  Statistically significant smaller change in the intervention group

*WB BMC/BMD* whole body BMC/BMD, *CTh* cortical thickness, *LS* lumbar spine. *Hip* total hip, *FN* femoral neck, *NR*: not reported

Dairy products consumption during childhood and adolescence leads to a higher peak bone mass, but data on statural height are less consistent [28]. In a recent systematic review, 8 out of 11 randomized trials performed during childhood and adolescence have revealed a 8% greater gain of BMD by 16 months of dairy products in various quantity [18]. A higher gain in lean mass with dairy products was reported in another meta-analysis [28].

One serving of dairies (30 g hard cheese, 2 dl milk or 1 yoghurt) represents 250 mg of calcium. Two servings are recommended below the age of 9 and 3 above, by various bodies in regions with Western style food habits [29, 30]. Three servings of dairies provide approximately 20 g of protein.

## Long-term effects of dairy products intake during childhood and adolescence

A high peak bone mass at the end of growth could theoretically contribute to a lower risk of fracture later in life [1]. It is estimated that a 10% higher bone mass could be equivalent to a menopause occurring 13 years later or to a 50% lower fracture risk [31]. However, though dairy products have been shown to increase bone mineral mass during growth, attempts to relate fracture risk in adulthood and aging, with dairy products consumption during childhood and adolescence, have not provided consistent results, likely in relation with the large inaccuracy of food intake recorded more than 40 years later. A frequent consumption of milk before the age of 25 years was associated with a higher proximal femur BMD between the age of 44 and 74 years [32]. A history of more than 1 glass of milk during childhood, but not during adolescence, compared to less than 1 glass per week, was associated with a higher trochanter BMD in postmenopausal women [33]. Less than one serving a day of dairy products during childhood was accompanied by a twofold higher risk of fracture in 50-year old women [34, 35]. In contrast, in the Health Care Professional study, no association was found between milk consumption during adolescence and hip fracture risk in women, with even a higher risk in men (+ 9% per additional glass of milk daily) [36]. This has been partially attributed to a greater height in dairy products consumers, higher height being a risk factor for hip fracture.

## Dairy products and bone mineral density and/or fracture risk in adults

### Calcium intake and fracture

The influence of calcium intake on bone remodeling and particularly on fracture risk has raised numerous debates for both antifracture efficacy and safety. Without entering the debate, the present evidence can be summarized as follows [37]. The combination calcium and vitamin D is associated with a modest decrease in fracture risk, particularly in the oldest old living in nursing homes [38]. Calcium alone does not appear to significantly influence fracture risk. Among the adverse events associated with calcium supplements, gastro-intestinal discomfort, more frequent with calcium carbonate preparations, and a slightly increased risk of renal lithiasis should be mentioned. A higher risk of myocardial infarction is not consistently confirmed, and is not present when calcium is from dietary origin, such as provided by dairy products [37, 39]. Similarly, accelerated vascular calcification, which can result from high pharmacological calcium supplementation, is not observed with calcium of dietary origin [11, 37, 39].

## Dietary protein and fracture risk (for review, see [40])

Numerous observational studies have addressed the issue of fracture risk in relation to dietary protein intake. The results of these studies are sometimes divergent. Positive associations, i.e. a higher fracture risk at high protein intake are rare, and are mostly observed with a low calcium intake [40]. In several systematic reviews and meta-analyses, hip fracture risk was lower with higher dietary protein (for review, see [40]), provided calcium intake is sufficient. It should be noted that there is no evidence of osteoporosis, changes in bone strength or in fracture risk in relation with dietary protein-derived acid load in a balanced diet [40, 41].

## Dairy products and bone remodeling

In short-term intervention trials (usually less than 4 months), dairy products reduced bone turnover markers by 6–40% together with a lowering of PTH levels in younger adults (Table 5) as well as in older individuals (Table 6). In a 12-week trial in overweighed adolescent girls, who were following a physical exercise program for weight loss, four servings of dairy products per day compared to two or less, decreased serum CTX [42]. This decrease was proportional to the number of servings. In 85-year-old institutionalized people, 2 servings/day of soft white cheese fortified with vitamin D and calcium during 6 weeks reduced PTH and bone resorption markers [92].

**Table 5:** Effects of dairy products on bone in younger adults (controlled trials)

Study	Year	Population	*N*	Mean age (years)	Intervention	Duration	Outcomes	Main results	Conclusions: effects of dairies
Baran et al. [90]	1990	Premenopausal women	37	~ 36	Dairy products equivalent to + 610 mg/day of Ca	3 year	PTH, LS BMD	PTH: no change; LS BMD:—0.4 vs—2.9% in controls	Prevention LS BMD loss
Bonjour et al. [91]	2008	Postmenopausal women	30	59.5	Semi-skimmed milk 500 ml/day	6 weeks	BTM, PTH	PTH:—3.2 pg/ml; CTX:—624 pmol/l; P1NP:—5.5 ng/ml; Osteocalcin:—2.8 ng/ml	↘ PTH, ↘ CTX, ↘ P1NP, ↘ Oc
Bonjour et al. [93]	2012	Postmenopausal women with low spontaneous supply of Ca and Vit D	71	56.6	2 servings of skimmed-milk and soft white cheese fortified with Vit D (2.5 μg/d) and Ca (400 mg/d)	6 weeks	IGF-I, BTM	IGF-I: + 18 µg/l; TRAP 5b:—0.3 U/l; CTX: NS	Greater ↗IGF-I and ↘ TRAP5b
Chee et al. [94]	2003	Postmenopausal (> 5 years) women (55–65 years)	173	59	Milk powder with 1200 mg/d Ca	24 months	BMD	LS BMD:—13 vs—90%; Hip:—0.50 vs—2.17%; FN BMD: + 0.51 vs—1.21% in controls	↗Vit D, ↘ spine and hip BMD loss, benefit still evident 21 months after the study end
Ting et al. [95]	2007	Postmenopausal (> 5 years) women (55–65 years)	173	61	Milk powder with 1200 mg/d Ca	24 months	BMD	Some difference still detectable 18 months after intervention end	
Chen et al. [96]	2015	Postmenopausal women	141	55.9	Milk powder with 900 mg/d Ca	24 months	BMD	LS:—013 T-score difference in favour of intervention group	↘ LS BMD loss
Gui et al. [49]	2012	Postmenopausal women without osteoporosis (45–65 years)	141	56.5	Milk/Soymilk with 250 mg/d Ca	18 months	BMD	Milk: Hip: + 2.5%; FN: + 2.8%. Soymilk: not different from controls	Prevention FN and Hip BMD loss
Josse et al. [102]	2010	Young women	20	23.2	500 ml skimmed milk before and 1 h after exercise	12 weeks	PTH, BTM	PTH:—1.2 pmol/l	↘ PTH
Josse et al. [103]	2012	Young overweight women	90	~ 31.5	6–7 servings/day dairy	16 weeks	PTH, BTM	PTH:—1.2 vs + 0.8 pmol/l; P1NP: + 16 vs + 1 µg/l; CTX: + 0.01 vs + 0.12 nmol/l in controls	Prevention of ↗ bone resorption
Kristensen et al. [104]	2005	Healthy young men (22–29 years)	11	24	2.5 l/day of Cola + low-Ca diet vs 2.5 l/day of semi-skimmed milk + low-Ca diet	10 days	BTM	CTX: 0.8—> 0.6 with milk vs—> 0.9 with cola	↗ BTM with cola diet, not milk diet
Kruger et al. [105]	2006	Premenopausal women 20–35 years	82	27	High Ca skimmed milk (1000 mg/d of extra Ca)	16 weeks	BTM	sCTX: 0.49—> 0.30 ng/ml; P1NP: 55.9—> 42.1 ng/ml	↘ CTX, ↘ osteocalcin, ↘ P1NP
Kruger et al. [106]	2010	Postmenopausal women	120	57.5	Milk powder fortified with 1200 mg Ca, 96 mg magnesium, 2.4 mg zinc and 9.6 μg Vit D /d	16 weeks	Vit D, PTH, BTM	CTX:—40%; osteocalcin:—30%; P1NP:—30%	↘ BTM
Lau et al. [109]	2001	Postmenopausal women	200	57	Milk powder providing 800 mg/day Ca and 18.8 g protein	24 months	BMD	Hip:—0.06 vs—0.88%; LS:—0.56 vs—1.5%; FN:—0.70 vs—1.1% in controls	lower ↘ BMD, ↗ Vit D, ↘ PTH
Lau et al. [110]	2002	Postmenopausal women	187	57	Milk powder containing 800 mg/d Ca	36 months	BMD	Lower BMD loss; Hip 81%; LS: 65%; FN: 73%	lower ↘ BMD
Liu et al. [111]	2011	Pregnant women (24–31 years) with habitual low Ca intake	36	27	Milk powder (containing 350 mg Ca); milk powder (containing 350 mg Ca) + 600 mg Ca/d	20 weeks gestational age to 6 weeks post-partum	BMD, BTM	Higher WB and LS BMD in the milk high calcium group	↗ BMD
Moschonis et al. [113]	2010	Postmenopausal women (55–65 years)	66	60	Milk and yogurt fortified with 1200 mg Ca and 7.5/22.5 μg Vit D + counselling	30 months	BMD	WB BMD: + 0.003 vs—0.020 g/cm2 in controls; spine: + 0.118 vs + 0.049 g/cm2 in controls	↗ WB BMD whole body and spine
Recker et al. [116]	1985	Postmenopausal women	22	NR	192 ml/day milk	24 months	Ca balance	Ca balance:—0.061—>—0.017 g/day	Better Ca balance
Rosado et al. [117]	2011	Young obese women	139	34	3 × 250 ml/day low-fat milk	16 weeks	BMC	WB BMC: + 28 vs—2 mg in controls	↗ WB BMC
Tenta et al. [119]	2011	Postmenopausal women	40	55–65	Milk and yogurt fortified with Ca (1200 mg/day) and Vit D (7.5–30 μg/day)	30 months	BTM, BMD	RANKL:—0.08 vs + 0.01 pg/ml; CTX:—0.11 ng/ml by 12 months	Prevention ↘ Vit D in winter. ↘ CTX and RANKL; ↗ WB BMD
Thorpe et al. [120]	2008	Overweight men and women (30–65 years)	130	46	1.4 g/kg BW protein through 3 servings/day of dairies	12 months	BMD	BW at 12 months:—10.5% in both groups. WB, LS and Hip BMD 1.6, 2.1 and 1.4% higher	↘ BMD decrease
Woo et al. [122]	2007	Women (20–35 years)	408	28	Milk powder with 1000 mg Ca, 80 μg Vit K	24 months	BMD, BTM	Overall, small BMD increases	No difference between groups

*NR* not reported, BMD bone mineral density, *BMC* bone mineral content, *WB* whole body, *LS* lumbar spine, *FN* femoral neck, *Ca* Calcium, *BTM* bone turnover markers, *Oc* osteocalcin

**Table 6:** Effects of dairy products on bone in older adults (controlled trials)

Study	Year	Population	N	Mean age (years)	Intervention	Duration	Outcomes	Main results	Conclusions: effects of dairies
Bonjour et al. [92]	2009	Institutionalized women ≥ 65 years old with low Vit D status and Ca intake < 700 mg/d	37	84.8	2 servings of soft white cheese fortified with Vit D (+ 1.25 µg/100 g) and calcium (total Ca 151 mg/100 g)	6 weeks	PTH, BTM	CTX:—7.5%; TRAP 5b:—9.9%; P1NP: + 19.3%; PTH:—12.3%; IGF-I: + 16.9%	↗ Vit D, ↗ IGF-I, ↘ PTH,
Daly et al. [97]	2005	Men (50–79 years) without Vit D deficiency	167	62	400 ml/day fortified with 1000 mg calcium and 800 IU vit D	24 months	BMD, PTH, vit D	2 years: FN:—0.7 vs- 2.22%; UD radius:—0.71 vs—2.28%; first year: 25OHD: + 31%; PTH:—18%	↗Vit D, ↘ FN and UD radius BMD loss
Daly et al. [98]	2008	Community living men (50–79 years)	111	63	Fortified milk with Ca (1000 mg/d) and Vit D (800 IU/d)	18 months	BMD	Some difference still detectable 18 months after intervention end	
Green et al. [99]	2002	Postmenopausal women	50	67.5	Milk powder fortified with 1200 mg calcium	4 weeks	PTH, BTM	sCTX: 0.43—> 0.28 ng/ml	↘ CTX
Heaney et al. [100]	1999	Men and women, 55–85 years, less than 1.5 serving/day dairy	204	65.1	3 servings/day of low–fat milk	12 weeks	IIGF-I, urine NTX	uNTX:—13%; IGF-I: + 10%; IGFBP4: stable whilst + 7.9% in controls	↗ IGF-I, ↘ uNTX
Heaney et al. [101]	2002	Postmenopausal white women with Ca intake < 600 mg/d	29	61.4	3 servings/day of yogurts	7–11 days	Urine NTX	uNTX:—22%	↘ Urine NTX
Kruger et al. [107]	2012	Postmenopausal women	63	62	Milk fortified with 900 mg Ca, 96 mg magnesium, 2.4 mg zinc and 6.4 μg Vit D /d	12 weeks	Vit D, PTH, BTM	PTH:—14%; CTX:—29%; P1NP:—18%	↗ Vit D, ↘ PTH, CTX, Oc, P1NP
Kukuljan et al. [108]	2009	Men (50–79 years) without Vit D deficiency	180	61	400 ml/day milk fortified with 1000 mg/d Ca and 800 IU/d Vit D ± exercise	12 months	BMD	LS: + 1.5% vs controls; Hip: + 0.7% vs controls	No interaction with exercise
Manios et al. [112]	2007	Post-menopausal women	101	61	Milk and yogurt fortified with 1200 mg Ca and 7.5 μg Vit D + counselling	12 months	IGF-I, BTM	IGF-I: + 38%; CTX: -23.2%; WB BMD: + 1.5 vs—0.7% in controls	↗ WB BMD whole body and spine; ↘ CTX; ↗ IGF-I
Moschonis et al. [114]	2011	Postmenopausal women	63	62	Milk and yogurt fortified with 800 mg Ca + 10 μg Vit D & Vit K	12 months	BMD	WB BMD: + 0.013 vs—0.001 g/cm2 in controls; LS: + 0.006 vs—0.032 g/cm2 in controls	↗ WB and spine BMD
Prince et al. [115]	1995	Postmenopausal women	84	63	208 ml/day milk with 1000 Ca	24 months	BMD	Trochanter: + 0.2 vs—0.6% per year, distal tibia:—1.5 vs—2.5% in controls	lower ↘ BMD
Storm et al. [118]	1998	Postmenopausal women	60	71	Milk 4 × 240 ml /day	24 months	BMD, BTM	Trochanter:—0.009 vs—0.022 g/cm2 in controls	↘ BMD decrease
Tu et al. [121]	2015	Men and women	65	66	1.6 l/day Kefir fortified with 1500 mg Ca	6 months	BTM, BMD	No difference between groups	No difference between groups

*BMD* bone mineral density, *BMC* bone mineral content, *WB* whole body, *LS* lumbar spine, *FN* femoral neck, *Ca* Calcium, *BTM* bone turnover markers, *Oc* osteocalcin

## Dairy products and bone mineral density

In a meta-analysis including 20 studies and 37,174 subjects, lumbar spine and femoral neck BMD was lower in subjects avoiding any dairy product, like vegans, than in vegetarians, thus a diet without meat and fish but including dairy products, as well as in omnivores [43]. In a meta-analysis evaluating the role of dietary patterns on prevalence of low BMD, a diet rich in dairies was associated with a 41% lower prevalence of low BMD [44].

In a randomized trial assessing the effects of a calcium–vitamin D supplement on BMD in men and women older than 65 years, a positive association with dietary protein intake was observed, but only in the calcium–vitamin D-treated group [45]. This suggests a possible interaction between dietary calcium and protein [3]. Various intervention trials with milk powder, dairy products fortified in calcium or vitamin D, lasting between 5 and 30 months, have shown an attenuation of the age-related bone loss (Tables 5 and 6). As a possible mechanism of action of dairy products on bone strength, a tibia diaphysis cross-sectional area proportional to the number of serving of dairies has been reported [46]. Integrating values of bone microstructure to estimate bone strength, finite element analysis has revealed higher values of distal radius and tibia failure load in relation with dietary protein of dairy origin in both 65-year-old women [47] and in 84-year-old men [48]. In both studies, carried out in different populations of different sex and age, there was no significant association of bone failure load with protein of vegetable origin. A randomized controlled trial in 141 postmenopausal women has concluded that the consumption of cow milk was superior in preventing BMD loss at the hip and femoral neck over an 18 months period compared to soy drink [49] (Table 5). Although the calcium intake was similar in both groups, the observed skeletal differences were attributed to a potentially higher bioavailability of calcium from milk. In a meta-analysis including 618 participants from 6 trials, there was a significant effect of dairy products on BMD, with effect size of 0.21, 0.36 and 0.37 for lumbar spine, femoral neck and total hip, respectively [50].

## Dairy products and fracture

In the same meta-analysis quoted above [43], the risk of any fracture was 44% higher in subjects avoiding any dairy product, like a vegan diet, as compared with a omnivore diet. The 25% higher fracture risk observed in vegetarians did not reach a level of statistical significance. These results suggest that a diet devoid of dairies could be associated with a higher fracture risk.

In the absence of controlled intervention trials with fracture as outcome, one should rely on observational studies, which have sometimes not provided consistent conclusions. In a 32-year follow-up of 123,906 subjects of both sexes, 1 serving of 240 ml of milk was associated with a 8% reduction of hip fracture risk. The reduction amounted to 6% per serving of any dairy products [51]. In two cohorts in Norway, a country with an usual high dietary calcium intake, which included 613,018 and 252,996 person-year, there was no association between hip fracture risk and milk consumption, with hazard ratio varying between 0.97 and 1.02 [52]. Not too far away, in Sweden, milk consumption up to six glasses of milk (200 ml glasses) was associated with a higher risk of hip fracture, but not of all fractures together, in a cohort of 61,433 women followed over 20 years, but not in men 45,339 men over 11 years [53]. Interestingly, in the same study, any serving of fermented dairy products, i.e. 200 g of yoghurt or 20 g of cheese, led to a 10–15% lower hip fracture risk, in women and in men [53]. Several recent meta-analyses have included various cohort or case–control observational studies assessing the relationship between hip fracture risk and dairy products consumption [3, 54–57]. Not only according to the number of studies included, but also according to the subjects origin and the type of dairy products, the results may vary, with differences in hip fracture risk reaching or not a level of statistical significance (Table 7). Overall, a lower hip fracture risk varying between 13 and 32% was found in dairy products consumers in some analyses, particularly with fermented dairy products. Thus, while the association between hip fracture risk and milk consumption is not fully consistent, the inverse relationship with fermented dairy products, particularly yoghurts, is more often reported (Table 7). However, during the menopause transition, fracture risk was not influenced by dairy products, probably in relation with the low number of events [58].

**Table 7:** Hip fracture risk in relation with dairy products consumption in recent meta-analyses

Meta-analyses	Studies		Hip Fracture		
		Milk	Yoghurt	Cheese	All dairies
Bian et al. 2018 [54]	Cohorts (10)	0.91	0.75*	0.68*	0.87*
	Case–control (8)	0.71*	0.77	0.77	0.75*
Matia-Martin et al. 2019 [55]	Cohorts (5)	0.91	0.87	0.80	0.87
Malmir et al. 2020 [57]	Cohorts (14)	0.93			0.90
	Case–control (9)	0.75*			0.86
Hidayat et al. 2020 [56]	Cohorts (9)	0.86	0.78*	0.85	
	In USA	0.75*			
	In Scandinavian countries	1.00			
Ong et al. 2020^§^ [65]	Cohorts (3)		0.76*	0.89	

*Bold value indicates statistically significant

^§^Fermented products only

## Fermented dairy products (for review see [59])

The highest number of cells and particles in the human body are located in the digestive tract, as commensal organisms, collectively called gut microbiota [60]. The latter varies with age, living conditions, diet and some drugs, including calcium and vitamin D. Agents produced and released by the gut microbiota influence intestinal endocrine function, epithelial permeability and the immune system. Variations in gut microbiota composition and function are implicated in a large series of various disorders such as intestinal, tumor, metabolic, auto-immune, inflammatory and neurologic diseases. Gut microbiota is also modified by prebiotics, which are non-digestible food components, such as fibers or oligosaccharides, which are fermented in the large intestine. Galacto-oligosaccharides contained in mother milk help to child growth and to the development of the immune system [61]. Probiotics are organisms which, when ingested in sufficient amount, can influence intestinal content metabolisms. In human, one of the sources of probiotics is fermented dairy products, like yoghurts, fermented milk and cheese. One yoghurt serving contains about 10 million bacteriae (*Lactobacillus bulgaricus et Streptococcus thermophilus*). Dietary calcium could modify gut microbiota by favoring the proliferation of lactobacilli [62].

**Fig. 1:**
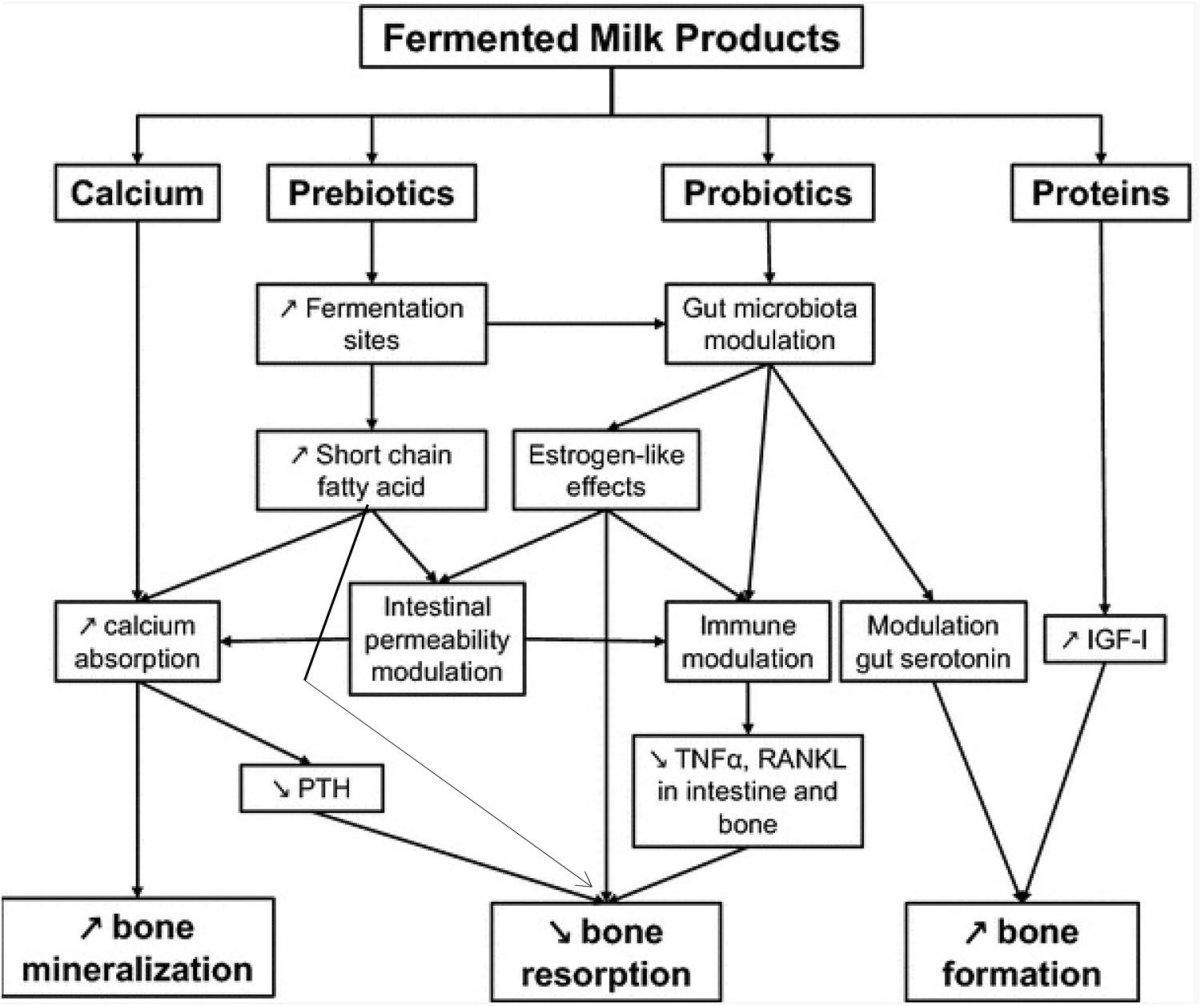
Influence of fermented dairy products on bone metabolism. Adapted from [59] with permission from the publisher. Fermented dairy products provide calcium, protein, prebiotics and probiotics, which could favorably influence bone remodeling by acting through different pathways

In adults, consumption of fermented dairy products attenuates age-related bone loss [59]. In a cross-sectional study in home dwelling subjects older than 60 years, yoghurts ingestion was associated with better bone mineral mass and muscle function [63]. For one serving of yoghurt, the risk of osteoporosis was 40 and 50% lower in women and men, respectively. In 65-year-old healthy women, peripheral skeleton cortical bone loss was inversely correlated to yoghurt intake frequency [64]. Short-term intervention trials have shown that yoghurt or cheese consumption reduced PTH and biochemical markers of bone resorption, without affecting bone formation markers [59, 65] (Tables 5 and 6). The effect of fermented dairy products on bone metabolism are summarized in Fig. 1. In a meta-analysis of 3 cohorts including 102,819 subjects, yoghurt consumption was associated with a 26% reduction in hip fracture risk [65] (Table 7).

## Tolerance to dairy products and safety

The milk of ruminants contains around 5% lactose, a disaccharide composed of glucose and galactose (Table 2). To be absorbed, lactose has to be hydrolyzed by the enzyme lactase. Individuals homozygous for CC alleles in the lactase gene are not able to digest lactose, and tend therefore to consume less milk as compared with lactase persistent subjects, because of symptoms of lactose intolerance like flatulence, abdominal pain and diarrhea, resulting from the fermentation of undigested lactose in the large intestine. A meta-analysis comparing lactose absorbers to lactase-deficient subjects, as determined by genetic testing or breath hydrogen test, in five case–control studies, has not found a difference in areal BMD [66]. However, when expressed in *Z* score, i.e., age-adjusted, lumbar spine and total hip displayed lower BMD values in lactase-deficient subjects. Lactase persistence and lactase non persistence did not differ in terms of hip fracture risk [67].

Another cause of intolerance to cow milk is the presence of A1 beta-casein, produced by some cow breeds, particularly those of European origin, instead of A2 beta-casein, found in Asian or African cattle [68]. Both beta-casein proteins, which represent 30% of total protein content in cow milk, differ by only one nucleotide changing the codon in position 67 of the 209 amino acid protein, with a change of histidine to proline. A1 but not A2 beta-casein digestion produces beta-casomorphin-7, which activates µ-opioid receptors located along the gastro-intestinal tract, and may account for an increase in gastro-intestinal transit time and abdominal pain. In a randomized, double-blind, cross-over trial, A1 beta-casein was associated with worst post-dairy abdominal discomfort, higher concentrations of inflammation-related biomarkers and lower levels of short chain fatty acids, as compared to A2 beta-casein [69]. Digestion of A2 beta-casein is easier. Beta-casomorphin-7 may be hydrolyzed by bacteria present in yoghurts during the fermenting process [70]. Whether casomorphins are implicated in the modified brain activity in regions that controls the processing of emotion and sensation in healthy women with a 4-week intake of fermented dairy products, is not known [71].

Dairy is a major source of saturated fatty acids. Previous meta-analyses, on which many dietary guidelines are based, have considered saturated fatty acids as associated with increased risk of cardiovascular diseases [72]. However, recent studies have indicated that all saturated fatty acids do not exert the adverse effect on cardiovascular disease as previously believed, and that the various saturated fatty acids exert very different biological effects, which are dependent on the food matrix [3, 73, 74]. For instance, cheese could be expected to increase cardiovascular disease risk because of its high content of saturated fatty acids and sodium, but observational studies indicate in fact a reduction in blood pressure and lower risk of cardiovascular disease and stroke with increased cheese consumption [3, 75]. Dairy fat eaten in the form of cheese affected blood lipids differently from when the same constituents were ingested in different matrices [76]. Total cholesterol levels were even lower when all fat nutrients were eaten in cheese matrix. An updated meta-analysis including 29 cohort studies found inverse associations between total intake of fermented milk products, including soured products, yoghurt and cheese, with mortality and risk of cardiovascular disease (relative risk for both: 0.98) [77]. Neither plain milk nor low-fat milk were related to any increased risk of cardiovascular events. Risk of cardiovascular disease decreased by 2% per 10 g of cheese consumed per day. In a large cohort study of individuals aged 35–70 years enrolled from 21 countries in 5 continents, higher intake of total dairy (> 2 servings per day compared with no intake) was associated with a lower risk of total mortality, non-cardiovascular mortality, cardiovascular mortality and stroke. Higher intake (> 1 serving vs no intake) of milk and yoghurt was associated with lower risk of a composite outcome of the above events [78]. Finally, intake of whole fat yoghurt or cheese in place of milk was associated with a lower risk of myocardial infarction during a median 15.9-year follow-up [79].

## Conclusions

Among various nutrients, calcium and protein are of major importance for bone health. These nutrients are provided by dairy products. The latter contribute to meet nutrients needs. Intervention studies have shown beneficial effects of dairy products on bone mass accrual in children and adolescents, and on bone turnover in young and older adults. In observational studies, dairy products, particularly those fermented appear to be associated with a lower hip fracture risk.
